# Recent Advancements in Metal-Free Carbon-Based Catalysts: A Challenge in Sustainable Fine Chemical Synthesis

**DOI:** 10.3390/nano16110684

**Published:** 2026-06-01

**Authors:** Elena Pérez-Mayoral, Mounia Al Bahri, Ines Matos

**Affiliations:** 1 Departamento de Química Inorgánica y Química Técnica, Facultad de Ciencias, Universidad Nacional de Educación a Distancia (UNED), Urbanización Monte Rozas, Avenida Esparta s/n, Ctra. de Las Rozas al Escorial Km 5, 28232 Madrid, Spain; malbahri@ccia.uned.es; 2 LAQV, Associated Laboratory for Green Chemistry, Chemistry Department, NOVA School of Science and Technology | NOVA FCT, 2829-516 Caparica, Portugal

**Keywords:** carbon catalysts, fine chemicals, sustainable chemistry, metal free catalyst

## Abstract

The use of metal-free porous carbon catalysts can be considered one of the best alternatives for implementing a more sustainable fine chemical synthesis, as a challenge necessary to protect both our planet and society. It is recognized that the selection of the appropriate functional carbon catalyst, operating under the optimal reaction conditions, undoubtedly improves both the conversion and selectivity of a great variety of distinctive organic transformations, often through cascade reactions or even multicomponent synthesis. Increasing our knowledge of synthetic methodologies for metal-free carbon-based materials (including many of them on a large scale or even at an industrial scale), available characterization techniques, and computational methods represents an excellent opportunity to make these types of materials promising catalysts for a more sustainable future. In this context, this review is addressed to revisit the benefits and limitations of using each type of metal-free porous carbon catalyst in fine chemical synthesis, particularly in multi-bond forming processes for the synthesis of relevant heterocyclic systems.

## 1. Introduction

Contemporary lifestyles are intimately linked to environmental pollution, which shows a direct impact on ecosystems, and, hence, there are important implications for animal and human health. Environmental protection involving society, institutions, and governments is one of the challenges for a more sustainable future in which the use of chemistry and engineering to develop green practices can contribute to achieving Sustainable Development Goals (SDGs) [1].

In recent times, carbon materials have acquired relevance for numerous environmental solutions, especially as a type of catalyst that is attractive for green energy transition. Classical carbon-based catalysts have been successfully applied in catalysis for a long time [2]. However, this type of amorphous or disordered carbon materials—activated carbons (AC), carbon aerogels, carbon fiber, etc.—is often characterized by low activity, stability, and oxidation resistance [3]. With the development of advanced carbon nanomaterials—carbon nanotubes (CNTs), graphene-based materials, and 3D porous nanocarbons—with enhanced catalytic properties, both activity and selectivity, carbon catalysts have gained significant attention, acquiring a special relevance from the economic, technological, and sustainable points of view. In fact, carbon catalysts constitute an excellent option for sustainable energy conversion [4] and storage technologies [5] due to their great versatility and exceptional properties such as high surface area, tunable pore architecture, good electrical conductivity, chemical and mechanical stability, and, sometimes, also low cost.

Concerning the fine chemical synthesis, a great plethora of porous carbon catalysts from different origins and with diverse chemical compositions and textural properties have been successfully applied, even if carbon catalysts are not the most popular ones in this application field [6,7,8,9,10].

Nowadays, metal-free carbon catalysts, also known as carbocatalysts, have been acquiring particularly enormous attention in electric-, solar- and thermal-driven reactions [11]. In this context, carbon-based materials often present low catalytic activity, and, hence, they are generally used as catalyst supports. Thus, surface functionalization is required to create active sites and increase both activity and selectivity. The most used approaches for this purpose consist of originating defects or the incorporation of heteroatoms (N, S, and P, among others) at the surface, which modify the charge density of carbon, improving the adsorption of the reactants. The combination of experimental and theoretical mechanistic studies is essential in order to determine the active sites and understand how they work, but also to serve as a guide for the design and development of new and more efficient carbon-based catalysts [12,13].

Metal-free porous carbon-based catalysts then emerge as an interesting sustainable alternative for fine chemical synthesis, offering the opportunity to study the nature and reactivity of the active sites in depth. The use of metal-free carbon catalysts in key organic transformations offers numerous advantages, including well-defined and highly active sites due to their high surface area, as well as improved stability because of the controlled shape and size, and, hence, enhanced catalytic performance. These materials have been extensively investigated mainly in selective oxidation–reduction reactions, including the oxidation of aromatic or aliphatic hydrocarbons and alcohols, the alkene epoxidation and reduction of nitroarenes, and alcohol amination and coupling reactions, but also in a reduced number of acid–base catalyzed reactions such as transesterification, hydrolysis, or Knoevenagel condensation [3]. This review aims to revise the synthetic strategies developed to produce metal-free carbon catalysts, considering the advantages and disadvantages, and their application in green fine chemical synthesis, specifically multi-bond forming processes in the liquid phase and under thermal-driven catalysis to synthesize heterocyclic systems by following a strategy of building the heterocyclic ring. Special attention will be paid to correlating the physico-chemical properties and surface chemistry of the catalysts with catalytic performance.

## 2. Synthetic Strategies and Precursors for Metal-Free Carbon Catalysts Synthesis

Carbons materials have been extensively studied in great variety of applications. In this sense, numerous interesting current reviews concerning this topic have been published [14,15,16,17,18]. Therefore, this section aims to highlight the carbon catalyst variability and provide a brief reminder of each type.

### 2.1. Carbons Catalyst Variability: Active Sites

**Figure 1:**
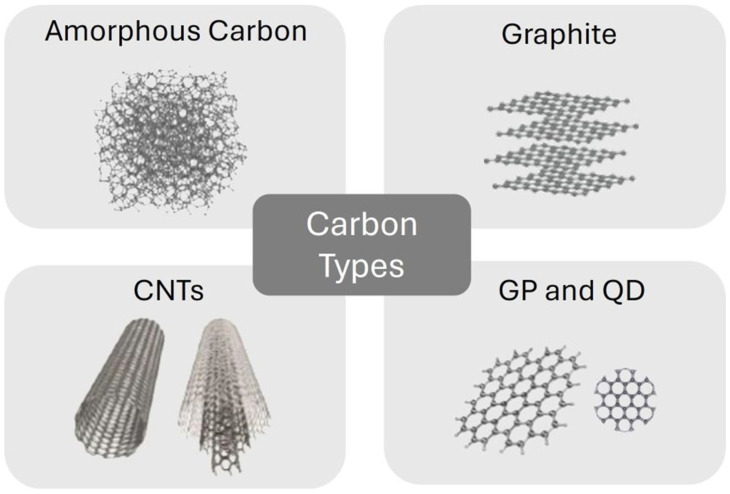
The main types of carbon catalysts.

Currently, there are many different types of carbon-based materials, with very different characteristics in structure, surface composition, morphology, and textural features, resulting in striking different properties and behaviors (Figure 1). Therefore, each type represents an opportunity, depending on its application. In addition, concerning sustainability, carbon-based materials are very versatile materials showing their environmentally friendly nature. There are important features that grant these materials their attractiveness in so many fields of application, namely, chemical and thermal resistance, physical stability, light weight, electrical conductivity and tunable porosity and surface chemistry [19]. Generally speaking, the main types of carbon materials can be identified as: Graphite, Activated Carbon (AC), Carbon Nanotubes (CNTs), Graphene (GP) and Carbon Fiber (CF).

Graphite is a layered carbon material composed of hexagonally arranged atomic planes, with this material being suitable for making electrodes, since it has good electrical and thermal conductivity. AC is an amorphous, disordered structure characterized by a highly developed pore structure and an exceptionally large specific surface area. There are a variety of synthetic procedures and they can be derived from a variety of raw material [20].

GP is a two-dimensional carbon material consisting of a one-atom-thick planar sheet of sp^2^ bonded carbon atoms arranged in a honeycomb pattern. It serves as the fundamental structural unit of several carbon allotropes: when wrapped, it forms fullerenes; when rolled, it becomes CNTs; and, when stacked, it forms graphite. Graphene oxide (GO) is a chemically modified form of graphene and is derived from graphite or GP through oxidation. During this process, oxygen-containing functional groups are introduced onto the graphene basal plane and edges. These groups disrupt the sp^2^ carbon network of pristine graphene, making graphene oxide highly hydrophilic and easily dispersible in water and many solvents [21].

CNTs, including single- and multiwalled carbon nanotubes, are one-dimensional nanomaterials known for their outstanding mechanical strength and excellent electrical and thermal conductivity. These materials are synthesized through well-established and commonly used chemical vapor deposition (CVD) processes, obtained from a carbon source and catalyst particles [22].

Other more recent forms of metal-free carbon catalysts include nanodiamonds (NDs) and nanodots, and a variety of other nanostructures such as nano-fibers, nano-coils, nano-horns, and nano-onions [23]. ND is another nanostructure of carbon that can be used as metal-free carbon catalysts after some modifications [24]. NDs can also be obtained by CVD; however, the most common synthetic route for commercially available NDs is the detonation approach [25]. While carbon dots are quasi-spherical carbon nanoparticles, amorphous or partially crystalline in nature, with sizes generally ranging from 2 to 10 nm, NDs are crystalline carbon nanoparticles characterized by a well-defined diamond lattice composed exclusively of sp^3^-bonded carbon atoms, ranging from 4 to 100 nm in size. Carbon dots possess a mixed sp^2^/sp^3^ carbon framework and are rich in surface functional groups such as hydroxyl (−OH), carboxyl (−COOH), and amine (−NH_2_), which contribute to their high chemical reactivity, good water dispersibility, and tunable surface chemistry. On the other hand, NDs present mechanical hardness and thermal stability, reflecting the intrinsic properties of bulk diamond, and the sp^3^ hybridization configuration infers some inertness, thus needing surface modification to improve their catalytic activity. Carbon quantum dots have also been reported as sustainable metal-free catalysts, for a wide range of organic transformations, catalyzing reactions such as oxidation reactions, biomass conversion, biodiesel production, C–C formation reactions, the aza-Michael addition, Knoevenagel condensation, Aldol reactions, among others [26].

**Figure 2:**
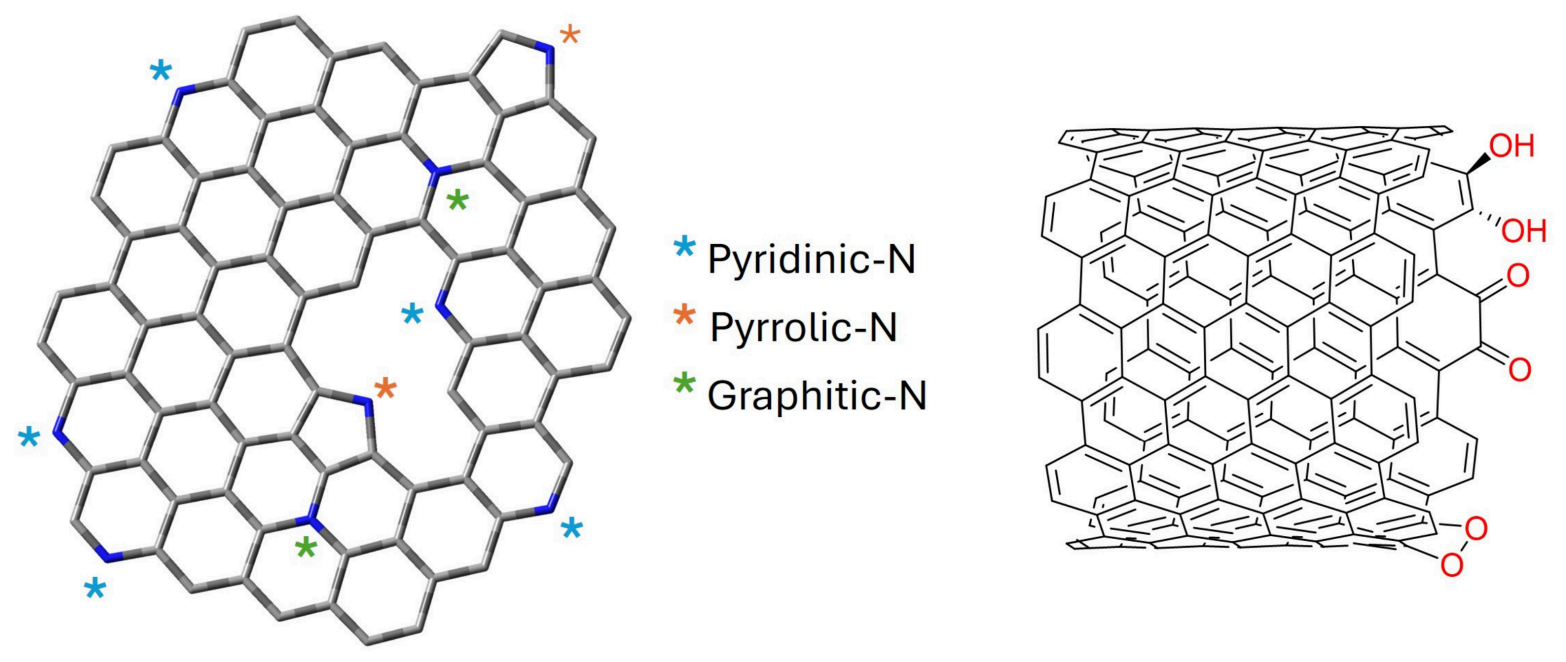
Example of functional groups in edges and defects in different morphologies.

**Figure 3:**
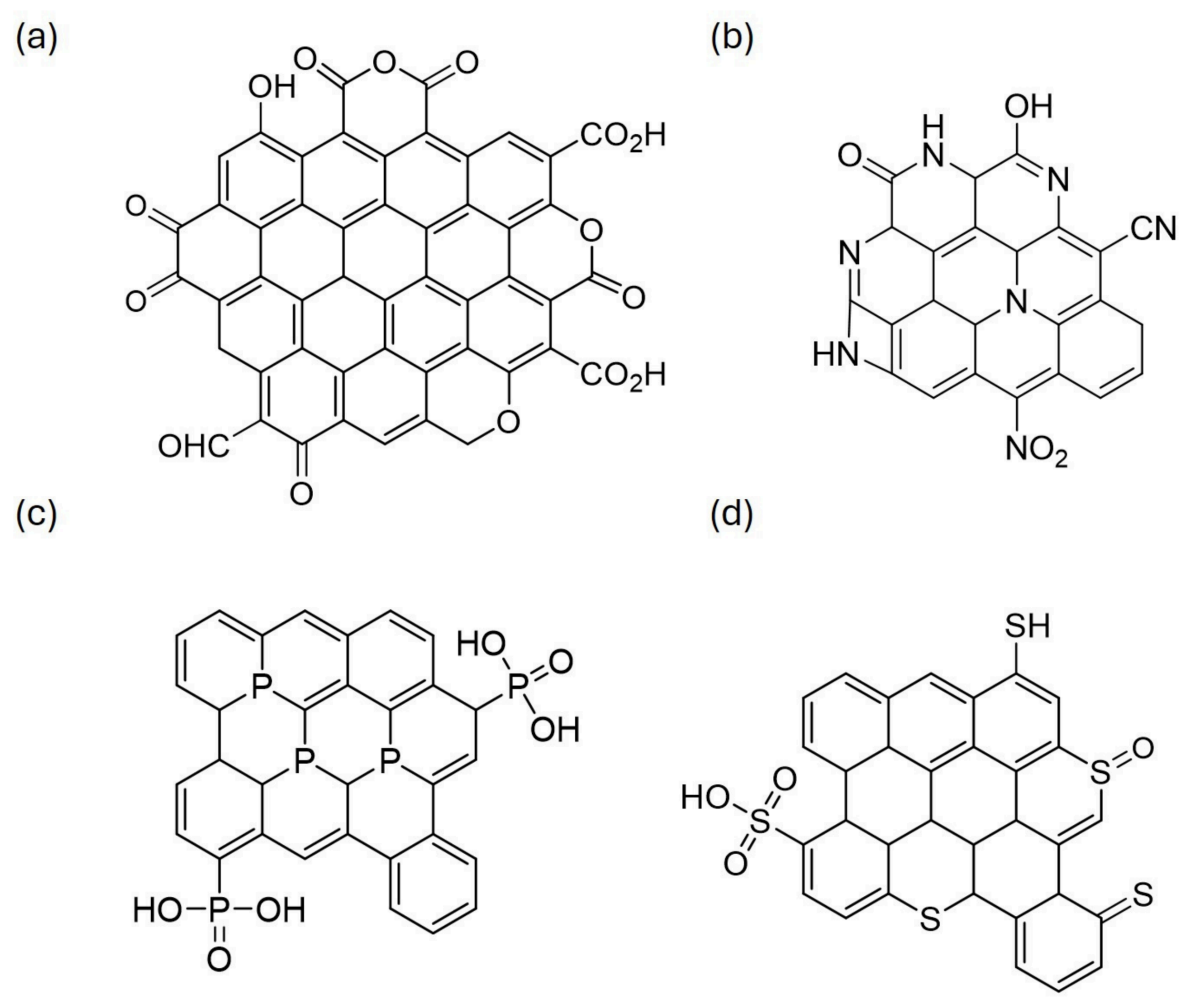
Active sites at the carbon surface depending on the dopant agent: (**a**) Oxygen, (**b**) Nitrogen, (**c**) Phosphorus, and (**d**) Sulfur.

For catalytic applications, it is of extreme relevance to the nature and type of functional groups present in the surface, edges, and defects of the materials. Besides carbon, which is the main element in the composition of these materials, there are other elements present to a much smaller degree. Oxygen, hydrogen, nitrogen, sulfur, phosphorus, and others are also present as heteroatoms and are responsible for some of the chemical characteristics of carbon materials. In fact, heteroatom doping has been exploited as a powerful and successful way to tailor the catalytic properties of the metal-free carbon catalysts (Figure 2 and Figure 3) [27].

Among the most studied functionalities on the carbon surface are the oxygenated surface groups (OSGs). Several OSGs have been identified and recognized on the surfaces of carbon-based materials in varying amounts: carboxylic acids, carbonyl, quinone, ether, phenol, and lactone. Other heteroatoms can be naturally present in carbon materials, due to the precursor nature or the synthesis method used, or they can be introduced by posterior doping the carbon materials with atoms such as nitrogen, boron, phosphorus, and sulfur [28].

Nitrogen is one of the most commonly studied dopants, primarily because its atomic radius closely matches that of carbon. This contributes to an easier integration in the carbon matrix with less disruption. Nitrogen is more electronegative than carbon, driving electron migration from carbon to nitrogen atoms, creating electron-deficient carbon sites adjacent to the nitrogen dopants, particularly in graphene-like materials [27]. Nitrogen incorporation may occur mainly in three distinct configurations: graphitic-N, pyridinic-N, and pyrrolic-N. Graphitic-N integrates into the carbon lattice by replacing a carbon atom within the hexagonal ring structure, while pyridinic-N and pyrrolic-N occupy edge or defect sites in the carbon structure.

The presence of Sulfur in carbon materials is also a common approach to introducing new features and characteristics to the material. Sulfur has a similar electronegativity to carbon, not imposing any charge transfer; however, due to the difference in atomic size, it creates a local distortion in the graphene structure. Additionally, XPS analysis has revealed the presence of C=S, C–S, –COS, and SOx functionalities on the ACs surface as well as the possible presence of S–O, C–O, C–S–C, and C–SH functional groups [29].

Phosphorous is another important dopant in carbon materials. It is in the same periodic group as Nitrogen but has a larger atomic size. The introduction of Phosphorous induces significant structural distortions and a local curvature in the graphene plane. In ACs, the Phosphorus can be present in the edges and defects of the graphitic structure, in the form of phosphorus-containing groups, and phosphate-like surface groups where the dominant linkages are C–O–P and C–P–O, accompanied by smaller amounts of C_3_–P=O species [30].

Finally, Boron has lower electronegativity (2.04), which results in electronic effects opposite to the ones observed with nitrogen: electron transfer occurs from carbon to boron creating partially positive boron sites that can serve as catalytic centers [31]. Boron can also act as a Lewis acid, promoting the adsorptive interaction of nucleophilic molecules.

It is common for carbon material to be doped with two or more heteroatoms, as illustrated by the following very different examples. By using chitosan and sodium lignosulfonate as precursors, it was possible to prepare an N,S co-doped biochar containing abundant heteroatom-related functional groups, including pyridinic-N, graphitic-N, thiophene-S, oxidized sulfur species, and OSGs such as carbonyl and hydroxyl functionalities [32]. When tested in the persulfate activation in the degradation of tetracycline, pyridinic-N, thiophene-S, and defect sites were identified as the primary active sites.

Haitao Hu et al. [33] reported the preparation and application of metal-free B,F co-doped nitrogen-rich carbon catalysts (BF-NCs) prepared using a hard template method using nano silica as a template and introducing heteroatoms in the synthesis step. By co-doping boron and fluorine into nitrogen-rich carbons, it was possible to obtain a good distribution of the heteroatoms through the porous structure.

On the other hand, AF 5 is a commercial carbon-based catalyst (Lewatit^®^ AF 5), that is a polymeric-derived porous carbon material with a high surface area and strong chemical resistance, engineered to have stable mechanical properties and tunable surface chemistry [34]. This material was modified by an acidic pre-treatment using hydrochloric, nitric, and sulfuric acids in order to modify its surface chemistry by introducing oxygen-, nitrogen-, and sulfur-containing functional groups. The catalytic performance of the modified AF 5 was evaluated during the oxidative atmospheric leaching of enargite. The enhanced catalytic activity was directly correlated with the presence of redox-active surface groups, particularly quinone-like and nitrogen-based functionalities, which facilitate the electron transfer and promote in situ oxidant generation; it was concluded that quinone groups are crucial for the catalytic role of AF 5 because they act as surface-bound redox mediators.

Zheng et al. [35] reported a metal-free carbon electrocatalyst synthesized via the polymerization of ionic liquid precursors followed by controlled pyrolysis. The catalytically active centers in this material are located at the hybrid sp^3^/sp^2^ carbon interfaces, where adjacent boron and nitrogen heteroatoms are coordinated. Electron transfer from boron to the neighboring nitrogen and carbon creates electron-deficient boron sites which serve as key catalytic centers.

### 2.2. Carbon-Based Catalysts from Different Precursors: Advantages vs. Disadvantages

The selection of the precursor is established as a critical variable for carbon-based catalyst design. The molecular constitution and structural architecture of the starting material exert a determining influence on the stoichiometry and morphology of the resulting carbonaceous material [36]. Specifically, the precursor defines the fundamental kinetic parameters of the catalyst, the porosity distribution, and the surface chemistry inherent to its purity and potentially for the control of the functionalization with heteroatoms [9]. This complex structural–functional interdependence dictates the intrinsic activity and catalytic selectivity required for successful organic synthesis reactions. Consequently, the following section presents a comparative and systematic analysis of the operational advantages and associated disadvantages of the two main precursor approaches: (i) from pure chemical substances, and (ii) from waste.

#### 2.2.1. Pure Chemicals

Among the metal-free carbon-based catalysts prepared from pure chemicals are the carbon gels–aerogels, cryogels, and xerogels–CNTs and carbon from some type of metal-organic frameworks (MOFs) and covalent organic frameworks (COFs).

Carbon gels prepared from the sol–gel method are highlighted by their extremely high surface areas, which maximize the dispersion of the active phase [37]. This high porosity, coupled with their low density and excellent thermal resistance, makes them ideal catalytic supports. The most common carbon materials derived from carbon gels are aerogels and xerogels. Aerogels synthesis presents the limitation of the necessity of using supercritical drying, which is the final and most critical step in their production. During this process, the original solvent is exchanged with supercritical CO_2_, preventing the presence of two phases, gaseous and liquid, and structural collapse [38]. In contrast, xerogels, obtained through more cost-effective atmospheric pressure drying, present the disadvantage of a dramatically reduced porosity and lower surface area, both key factors for catalytic applications [39].

On the other hand, CNTs stand out as advanced nanomaterials showing great potential mainly as catalytic supports overcoming the limitations of toxic and expensive metals. Their high mesoporous surface area and chemical inertness allow the direct and controlled functionalization of the surface with heteroatoms or organocatalytic groups by grafting [40], generating intrinsic active sites that confer a high selectivity in key reactions. However, their effective use faces significant operational challenges, the main one being the tendency to the agglomeration of the tubes due to strong Van der Waals interactions, which require functionalization or dispersion processes, often with a high cost to maintain the active surface. Furthermore, the difficulty in large-scale production and their isolation as metal-free remains a major obstacle.

Finally, carbon catalysts from MOFs and COF have also been investigated in fine chemical synthesis. The use of MOFs to prepare hierarchically pure carbons even doped with heteroatoms depending on the MOF compositions often requires high pyrolysis temperatures under inert conditions. Only a few specific metal-free carbons from MOF can be obtained by in situ reductive metal evaporation without additional treatment for leaching of metal species [41]. Otherwise, COFs are characterized by their customizable porosity and well-defined crystalline structures. This molecular architecture allows a homogeneous and controlled distribution of catalytic sites integrated into their network, which is essential for single-site catalysis. COF-derived carbon materials maintain the structural characteristics of their precursors such as porosity and hierarchical architecture, with an abundant concentration of defects and active sites [42]. However, the complexity of the COF synthesis represents significant limitations for their industrialization.

#### 2.2.2. From Waste

The transition toward a circular economy almost mandates the use of sustainable precursors or recyclables. The use of biomass and plastic waste as raw materials for the synthesis of carbon supports and catalysts not only addresses the need for renewable resources but also offers innovative solutions for global waste management [43,44]. However, the conversion of these heterogeneous and complex precursors presents an intricate balance between ecological advantages and the methodological and technical challenges that directly impact the performance of the final catalyst.

Biomass-nut shells, agricultural residues, etc. make up the precursor of choice for the synthesis of activated carbons and biochars. It is an abundant and renewable resource, ensuring a long-term supply and drastically reducing raw material costs. Its primary advantage lies in its inherent structure and chemical composition rich in carbon and oxygen, which, through processes like pyrolysis or hydrothermal carbonization, can generate carbon materials with a controllable porosity and surface area and a high density of OSGs. These functional groups act as anchoring sites for metallic species or as active sites. On the other hand, plastic waste (PE, PP, and PET) offers a promising pathway. Although they present challenges in separation and purification, their key advantage is their high carbon content and low ash proportion compared to most biomass sources. Furthermore, utilizing single-use or non-recyclable plastics for catalysis is a direct step towards large-scale waste valorization, transforming an environmental problem into a source of high-value-added materials.

Particularly, poly (ethylene terephthalate), commonly abbreviated as PET, is composed of polymerized units of the ethylene terephthalate monomer. It is the most common thermoplastic polymer resin in the polyester family. PET has a large number of applications, with most of the global production intended for synthetic fibers and the packaging segment. Since PET is a common synthetic polymer used in our daily lives for packaging water and carbonated soft drinks, it has become one of the most abundant municipal and industrial wastes. In terms of volume, PET accounted for approximately 6.2% of global plastics production in 2024 [45]. In addition, these wastes present a high strategic value to produce effective carbon support [46,47]. Common methods for utilizing PET waste are mechanical recycling, pyrolysis, and chemical transformation [48]. Among these methods, pyrolysis offers several advantages for the treatment of PET waste. Specifically, the char produced from PET pyrolysis has a high fixed carbon content and, after activation, a significantly higher specific surface area. Furthermore, pyrolysis can handle mixed or contaminated PET waste that is unsuitable for mechanical recycling, providing a more flexible and efficient waste management solution [49].

### 2.3. Synthetic Strategies for Metal-Free Carbon-Based Catalysts

This section summarizes the main approaches for metal-free carbon-based catalysts synthesis considering the functionalization of the surface to generate the active centers.

#### 2.3.1. Direct Synthesis, In Situ Synthesis, and Bottom–Up Synthesis

One possible approach to incorporate heteroatoms or active sites in carbon materials is by using heteroatom-rich precursors or reagents capable of encompassing such heteroatom into the final carbon material. These methodologies aim at the homogeneous incorporation of heteroatoms in the carbon matrix with high dispersion and high concentration. In this regard, for the production of the N,S co-doped porous biochar catalyst, the use of chitosan and sodium lignosulfonate as nitrogen and sulfur precursors, respectively, was proposed [32]. After the production of a cross-linked gel composed of the precursors, chitosan and lignosulfonate, the synthetic methodology included pre-cryocrushing, freeze-drying, and pyrolysis under an inert atmosphere. This allowed for the uniform incorporation of nitrogen and sulfur into the carbon framework while simultaneously generating a hierarchical porous structure. The direct carbonization of other heteroatom rich precursors such as COFs [50], biopolymers [51], or specific biomass residues has also been reported [52].

The direct carbonization of a carbon-rich precursor in the presence of a suitable doping agent is another common approach for biomass or residues derived metal-free carbon catalysts [53].

In another work [54], bifunctional S- and N-doped hydrochar catalysts were prepared via a one-step hydrothermal carbonization (HTC) process using glucose as the carbon precursor. The in situ heteroatom doping was promoted by urea and thiourea as nitrogen and sulfur sources, respectively, enabling their incorporation during carbonization.

In another sense, the CVD method is one of the methods used to produce nanocarbon structures. Depending on the metal catalysts and the reaction conditions, various types of structures can be obtained, featuring distinct defects and functional groups [55]. However, most of the functionalization of these nanomaterials is introduced through post-synthesis processes.

Carbon dot synthesis can be categorized into top–down and bottom–up approaches [26]. The bottom–up approaches are the most widely used and are preferred due to their simplicity, scalability, and greener chemistry. These approaches use small-sized molecular precursors, such as citric acid, glucose, urea, and ascorbic acid, as well as renewable carbon sources like biomass and bio-waste including fruit peels, sugars, leaves, nutshells, and polysaccharides. The most common synthetic procedures for this route are hydrothermal/solvothermal synthesis, thermal decomposition, microwave-assisted synthesis, and mechanochemical and pyrolytic routes. For carbon dots, top–down approaches involve breaking down bulk carbon materials into nanoscale dots. The most common methods reported for this strategy include laser ablation, electrochemical oxidation, ultrasonic treatment, and acidic exfoliation. Due to the higher energy demand and lower control over surface functionality, these methods are less frequently used [56].

#### 2.3.2. Post-Treatment Methods

Surface modification, post-doping, or top–down strategies aim at the tailoring of catalytic properties by controlling the amount and type of surface functional groups and defects, and their heteroatom composition over the carbon material surface. Often, these approaches lead to surface modification but do not reach their bulk properties. Many methodologies and techniques can be identified in the literature, with some of the most common ones consisting of acid and base treatments, impregnation, the grafting of ligands or complexes, electrochemistry [57], UV and microwave irradiation, and thermal treatments [58]. Many of these techniques are used to control the amount and type of OSGs [59]. The oxidation of the surface by an acid treatment, such as HNO_3_, H_2_SO_4_, and H_3_PO_4_, is a well-established procedure to increase the OSGs and the surface acidity [60].

Under the right conditions, other heteroatoms can be introduced on the surface, such as sulfur and phosphorous. In fact, sulfonation, achieved by treating carbon-based materials with concentrated H_2_SO_4_ or other sulfonating agents, has been reported to introduce strong Brønsted acid sites (−SO_3_H groups) onto the graphene surface, as well as other carbon structures [61]. Gas-phase oxidations are other methodologies of choice performed under controlled temperatures using CO_2_, O_2_, steam, and air as oxidants. Conversely, thermal treatment in ammonia atmospheres under high temperatures introduces nitrogen in the matrix and increases the basicity of the material [62].

Amine functionalization can represent another route to surface modification [63]. Through grafting, amine-containing molecules are covalently bonded to the surface, allowing new active sites and surface properties to be tailored. In this sense, the covalent bonding of other active phases can be found in the literature. Blandez et al. [64] reported the synthesis of different carbon materials with covalently anchored *N*-hydroxylphthalimide (NHPI), prepared by reacting the OH surface groups with the carboxyl acid substituent of NHPI to form an ester bond.

Another approach is the chloromethylation of the carbon surface followed by a posterior reaction with a suitable ligand [65]. Furthermore, the silanization reaction using organosilanes capable of reacting with OH groups of the surface makes it possible to graft different molecules, such as commercially available aminopropyltriethoxysilane (APTES), or cloropropyltriethoxysilane, or even designing a specific organosilane [66,67].

Composite materials are another expanding trend in the preparation of catalysts with tailored and multi-site catalysts. By combining Norit AC with saccharose through hydrothermal treatment, a composite structure consisting of the original porous Norit framework decorated with hydrothermal carbon derived from saccharose was obtained [68]. The enhanced catalytic performance was attributed to the synergistic combination of NORIT porosity and the acidic functionalities generated from saccharose-derived hydrothermal carbon.

From the above text, the number and diversity of approaches available to prepare metal-free carbon catalysts are apparent. Generally speaking, direct synthesis tends to result in a more uniform distribution of the doping element, and can favor co-doping, while post-treatment methods may result in less uniform materials, but favor surface modification, which may represent more accessible active sites. It is common for these materials to have more than one type of active site, or the same in different chemical environments. The understanding of the role of each specific catalytic site is an active topic of research in catalysis and is very much dependent on the reaction studied as well as its conditions. The inherent heterogeneity of the carbon materials hinders the perfect understanding of these mechanisms, and the challenge to obtain stable, tailored, and controlled metal-free carbon catalysts still remains.

## 3. Metal-Free Carbon-Based Catalysts in Sustainable Fine Chemical Synthesis

This section only refers to metal-free carbon catalysts reported for the synthesis of fine chemicals, through tandem reactions or in cascade processes, in the liquid phase, to produce heterocyclic systems of interest. These sustainable one-pot processes have gained importance, affording relatively complex molecules from multiple reactants, under green reaction conditions, by sequential reactions without the isolation of intermediate species, saving energy and operational time costs. They are often characterized by both high atom economy and efficiency, notably reducing waste formation, operating under green principles. In this case, carbon nitrides are excluded from this revision, addressed exclusively to thermal-driven catalysis as previously mentioned, since this type of material often works upon photochemical irradiation.

The catalytic behavior of metal-free carbon catalysts is mainly associated with the functionalization at the surface. OSGs classified as acid and basic oxygen functions, and often comprising other heteroatoms (mainly S, or P), are the most studied functions, although the carbon surface can also be modified by grafting with different functional groups in nature as mentioned above. The incorporation of these surface groups often confers a hydrophilic character to the carbon, favoring the high dispersion of these metal-free carbon catalysts in water, minimizing the environmental impact in the development of sustainable and more benign methodologies.

### 3.1. Classical Carbon Catalysts in Fine Chemical Synthesis

**Scheme 1:**
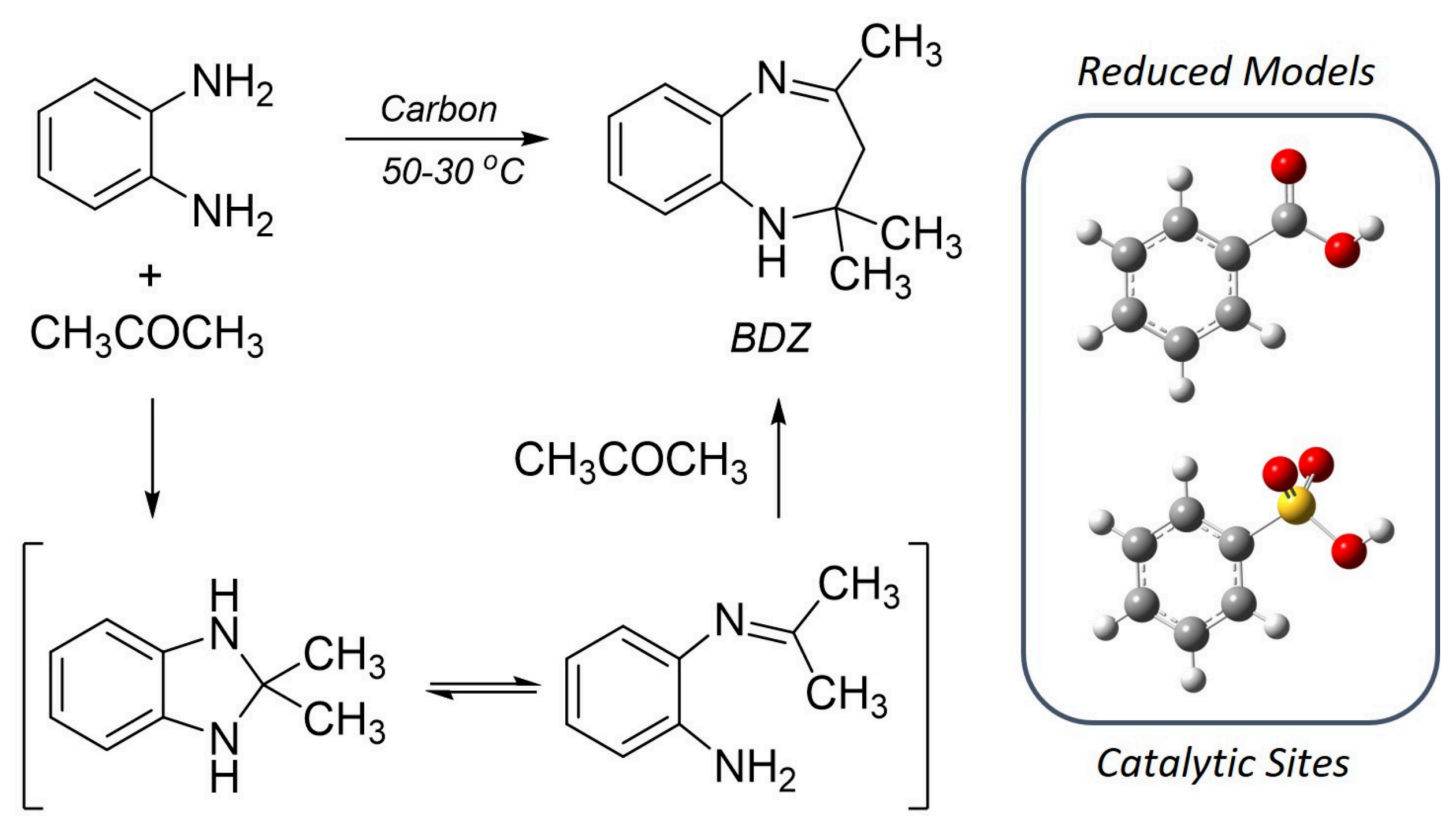
Synthesis of benzodiazepine (BDZ) from *o*-phenylendiamine and acetone catalyzed by acid porous carbons. H (white), C (grey), O (red), and S (yellow).

Metal-free ACs have been extensively studied in many varied applications; however, they are not the most investigated ones in fine chemical synthesis and even less so in tandem reactions. Among the most popular ACs are the functionalized C-SO_3_ with Brönsted acid sites, demonstrating high activity and reusability for industrially relevant organic transformations, and particularly in the synthesis of complex heterocyclic systems [69], as an alternative to other acid solids such as zeolites or sulfated metal oxides [70]. Synergistic effects between the chemical surface and the adsorption of the reactants on carbon lattice by non-covalent interactions such as p-p stacking and C-H-p hydrogen bonding are behind the superior catalytic performance. However, as far as we know, only a few examples of metal-free ACs showing Brönsted acidity have been documented in cascade reactions to synthesize heterocycles, correlating physico-chemical properties with activity, with our research group contributing to this progress. In this context, Godino Ojer et al. [71] reported a series of eco-sustainable and recyclable metal-free porous carbon catalysts to efficiently synthesize benzodiazepines (BDZs), under mild conditions, at 50 °C, with the aim to study the influence of the chemical surface and porosity in the selected process (Scheme 1). Porous carbons of our choice consisted of two different commercially available carbons—Norit RX3 (NORIT Netherland B.V., Klazienaveen, Netherlands) and a xerogel mesoporous carbon (Xerolutions S.L., Gijón, Spain)—and a laboratory-prepared ordered mesoporous carbon. The surface of all of them were modified by acid treatments using HNO_3_ or H_2_SO_4_ or both. Note that pristine carbons catalyzed the reaction with high conversion values (up to 70%, h), although with a low selectivity (40–60% approx.), whereas in the presence of oxidized samples, the selectivity is notably enhanced, especially for the samples treated with H_2_SO_4_, becoming 100% in the presence of the N-S catalyst. Among the samples treated with HNO_3_, the N-N catalyst, also derived from the Norit RX3 carbon, afforded the corresponding BDZ with both the highest conversion (up to 90%, 2 h) and selectivity (95%). Since this sample presented both the highest S_BET_ (1178 m^2^/g) and V_micro_ (0.54 cm^3^/g) but also the lowest amount of –CO_2_H groups, the obtained results are indicative of the great influence of microporosity. In addition, optimized transition structures, computed by using DFT calculations, of the key step consisting of the initial condensation between reactants in the presence of the most reduced models simulating the acid sites, such as benzoic and benzene sulfonic acid, revealed similar interaction modes. The hydrogen migration network was observed from the acidic proton from the corresponding model to C=O in acetone, with the C-N bond formation simultaneously taking place, followed by the H transference between the –NH_2_ groups, and, finally, restabilizing the acidic proton in catalytic active site. The combination of the experimental and theoretical results suggested that the formation and subsequent isomerization of the observed intermediate compound, as 2,3-dihydrobenzimidazole, is catalyzed by acid OSGs, with microporosity having a direct impact on selectivity.

**Scheme 2:**
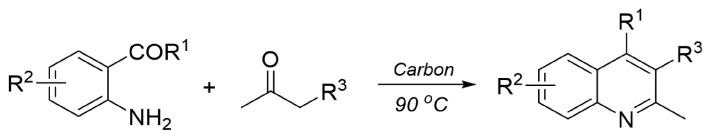
Synthesis of quinolines from 2-amino aryl ketones and 1,3-dicarbonyl compounds catalyzed by acid porous carbons. Copyright © 2013, John Wileyand Sons [72].

These porous carbons have been also active in the Friedländer reaction for the synthesis of quinolines with moderate to high conversions ranging 61–92% after 3 h (R^1^ = Ph, Me; R^2^ = H; R^3^ = COCH_3_) with total selectivity, superior to those obtained in the presence of other traditional acid catalysts such as BEA zeolite (27 and 60% after 5 h for R^1^ = Me and Ph, respectively), concluding that the microporosity and acidity of the surface are key factors determining the catalytic performance (Scheme 2) [72]. DFT calculations confirmed that the rate-limiting step, comprising the aldolic condensation between reactants, is favored by the presence of –SO_3_ groups on the surface. Optimized transition structures also revealed a series of simultaneous H migrations from (i) the catalyst to the forming alcohol, (ii) the enolic group to the amine function, and, finally, (iii) the H returning to the catalyst.

Much more recently, another series of porous carbon catalysts containing oxidized S- and P-OSGs, obtained from ZnO supported carbons using a fast acid-leaching strategy to alter the carbon surface by treatment with acids, in this case, H_2_SO_4_ or H_3_PO_4_, have also been investigated to synthesize BDZ with an almost quantitative conversion and total selectivity, after 2 h of reaction time, at 50 °C (Scheme 1) [73]. The reaction occurs even at lower temperatures (30 °C), although with a lower conversion and selectivity. Note that the catalyst nature is changed from a basic catalyst to the corresponding acidic ones, also modifying the porous structure. While the subsequent thermal treatment after acid treatment implies the release of –SO_3_ groups, the –PO_3_ functions were partially oxidized to –OPO_3_, with the latter showing a certain specificity in terms of increased selectivity. Therefore, both the concentration and acidic strength of catalytic sites condition the catalytic performance. The –SO_3_H functions at the surface particularly catalyze the final cyclization step, whereas the weak acidity of phosphorous species, –PO_3_H_2_ or –OPO_3_H_2_, in N3Zn-P samples promotes the cyclization more slowly, decreasing the selectivity. The high adsorption energy of BDZ on the –SO_3_H functionalized surface could be behind the conversion rate drop. Interestingly, the developed methodology to synthesize carbon catalysts has a direct impact on both the nature and distribution of acid sites on the surface. Such is the case for the NS and N3Zn-S catalysts, obtained from the commercial Norit RX3 sample and the corresponding ZnO-supported one by an acid treatment with H_2_SO_4_, respectively. While NS catalyst is mainly functionalized with –OSO_3_ groups, the predominant S-functions on the N3Zn-S catalyst are C-SO_3_, homogeneously distributed, and, notably, increasing the catalytic activity.

Godino-Ojer et al. [74] also reported a new macroporous carbon catalyst prepared by the pyrolysis of used tires and subsequently activated with H_3_PO_4_, efficient in the synthesis of BDZ, at 50 °C, in quantitative conversion after 4 h and high selectivity (90%) (Scheme 1). The catalyst presented a very low S_BET_ (48 m^2^/g), and the presence of phosphate functions and a silicophosphate (SiP_2_O_7_) phase, formed by the reaction of amorphous silica with H_3_PO_4_, was confirmed by XRD and XPS experiments. In this case, the most probable catalytic sites involved in the final cyclization step to BDZ consisted of acidic protons in pyrophosphate units at the edges as part of SiP_2_O_7_.

**Scheme 3:**
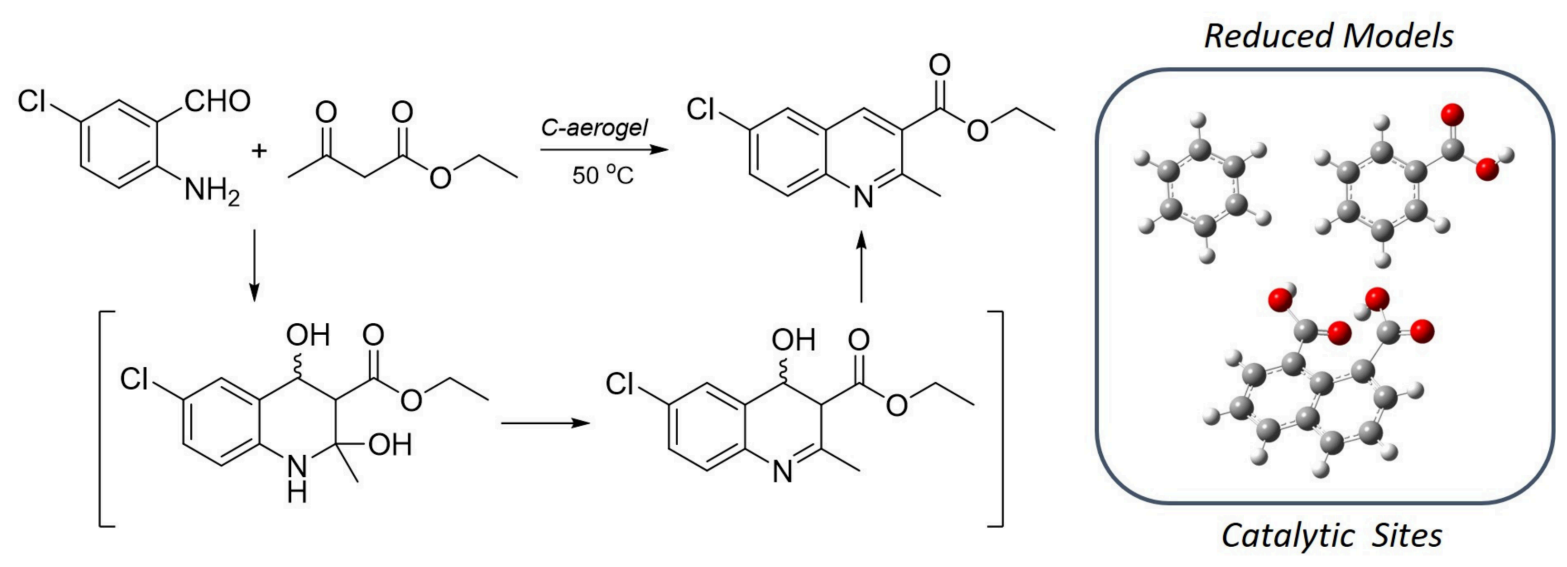
Synthesis of quinolines from 2-amino-5-chlorobenzaldehyde and ethyl acetoacetate catalyzed by acid carbon aerogels. H (white), C (grey), and O (red).

Other traditional pure porous carbons such as carbon aerogels prepared by resorcinol-formaldehyde polymerization, subsequently submitted to oxidizing treatment, have been applied for the synthesis of quinolines, via the Friedländer reaction, from 2-amino-5-chlorobenzaldehyde and ethyl acetoacetate, under microwave irradiation, notably reducing the reaction times (~40% of conversion after 4 h under thermal conditions at 50 °C vs. 66% of conversion after 15 min upon microwave irradiation) (Scheme 3) [75]. The investigated carbon aerogels showed both a high S_BET_ (up to 1000 m^2^/g) and developed microporosity and monomodal pore size distribution, showing mesopores (8 nm) or large macropores (~100 nm). The porosity was barely changed after oxidizing the carbon surface with (NH_4_)_2_S_2_O_8_ or H_2_O_2_. The oxygen content significantly increased for the samples oxidized with the stronger oxidant, (NH_4_)_2_S_2_O_8_, notably decreasing the pH_pzc_ from 10.3 to 4.3 and 3.1. Since some of the investigated samples present scarce oxygenated functions, the observed catalytic performance is probably governed by π,π stacking interactions between the reactants and the carbon surface. A more detailed study concerning the reaction mechanism and the active sites was reported, by DFT calculations, in the presence of different reduced models consisting of benzene, benzoic acid, and naphthalene-1,8-dicarboxylic acid. In this case, the operative reaction pathway is aldolization–cyclization–aromatization with double dehydration, confirming the interactions by π,π stacking even for the OSG-containing models. Models containing OSGs notably reduced the values of the relative free energy in all the elementary steps, with the carbon surface contributing to the activity mainly during the aldolization step through π,π stacking. The presence and concentration of –CO_2_H functions at the surface acting as individual catalytic sites and involved in each elementary step strongly favored the reaction.

**Scheme 4:**
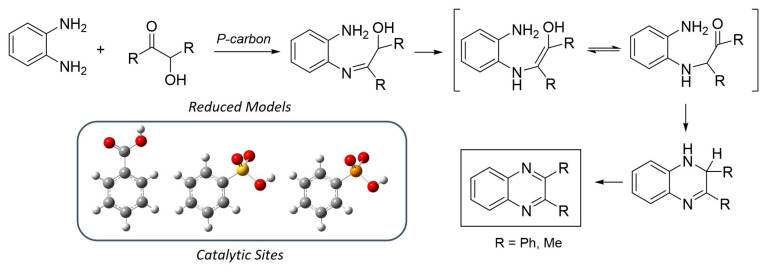
Operative route for the synthesis of quinoxalines from *o*-phenylendiamine and benzoin catalyzed by acid porous carbons. H (white), C (grey), O (red), P (orange) and S (yellow).

Phosphonated porous carbons obtained from vegetal biomass (*Hedychium gardnerianum*) by activation with H_3_PO_4_ in different amounts have also been reported for the efficient synthesis of quinoxalines in quantitative conversion and high selectivity (up 80%) (Scheme 4) [76]. These carbon catalysts showed a highly developed porosity; as an example, the RCB2 sample, prepared from a mass ratio of biomass to activating agent 1:3, presented a high S_BET_ (2197 m^2^/g) and high mesopore content with a 2.3–4 nm width. This sample showed different P species directly bonded at the carbon surface, CPO_3_ or C_2_PO_2_ groups, or even COPO_3_ or (CO)_3_PO functions with a bridge O atom, determined by XPS. Catalytic performance was also evaluated in the presence of other ACs with a different surface chemistry containing OSGs, such as –CO_2_H or acidic S-based functional groups, for comparison. In this case, the catalytic performance is governed by a combination of key factors, the acid strength of active sites, and textural properties in such a manner that a compromise between acidity and porosity resulted in an increment in both conversion and selectivity. The theoretical results revealed that the reaction mechanism consists of the cascade reactions involving the following reaction steps: imination reaction, successive imine-enamine and keto-enol tautomerisms, heterocyclization, dehydration, and final aromatization, with acid sites acting in each reaction step, whereas the adsorbed molecular oxygen on AC would be responsible for the last oxidative reaction. Remarkably, the energy barrier is notably decreased in the presence of acid sites compared with the uncatalyzed process, especially for the tautomerism steps.

### 3.2. Advanced Carbon Nanomaterials in Fine Chemical Synthesis

Among the advanced carbon materials, functionalized-GP and CNTs are the most investigated ones, highlighting GO as a highly oxidized form [77,78].

#### 3.2.1. Graphene-Based Catalysts

The high functionalization degree of GO and related catalysts makes them an eco-sustainable alternative to other metal supported catalysts, promoting a great variety of organic transformations, mainly oxidative and reduction processes, condensation, and coupling reactions [79,80].

##### Graphene Oxide (GO)

GO showing a soft acid character has been used as an efficient catalyst for the synthesis of a great variety of relevant heterocyclic scaffolds. Due to the hydrophilicity of GO, most organic transformations are described under greener conditions, favoring the use of green solvents such as water or alcohols in which GO is highly dispersed but also acting as a benign oxidant. In this context, different heterocyclic systems have been easily prepared in the presence of GO and summarized in Table 1, while other examples are commented on because of the valuable mechanistic information. Some details concerning the reaction conditions, as well as other heterogeneous catalysts for comparison, if any, often used in larger catalyst amounts, are also shown. When compared, the corresponding reaction in the presence of GO occurred more rapidly or afforded the corresponding heterocycle with a higher yield.

**Scheme 5:**
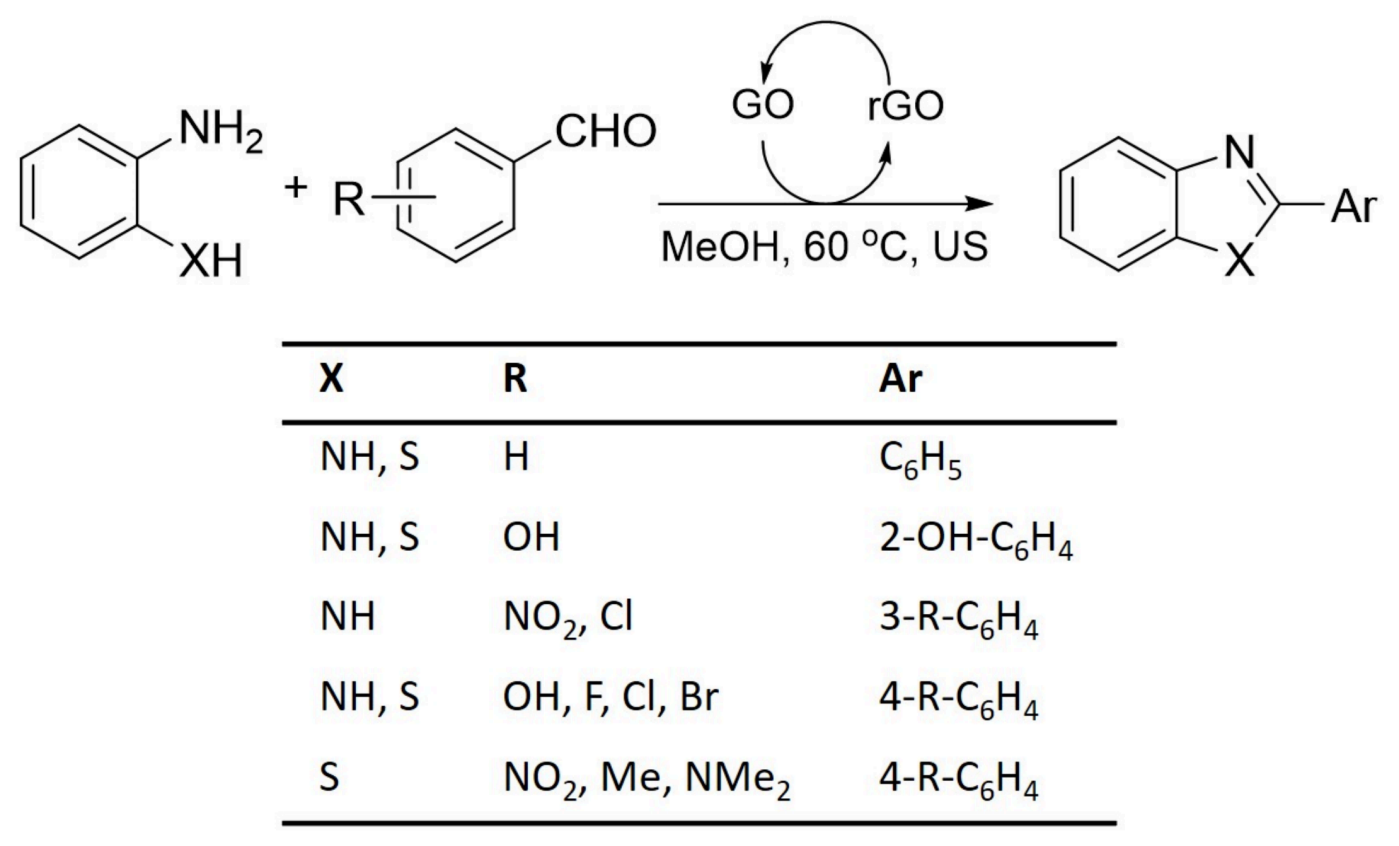
Synthesis of benzimidazole and benzothiazole from *o*-phenylendiamine and aromatic aldehydes catalyzed by GO.

**Scheme 6:**
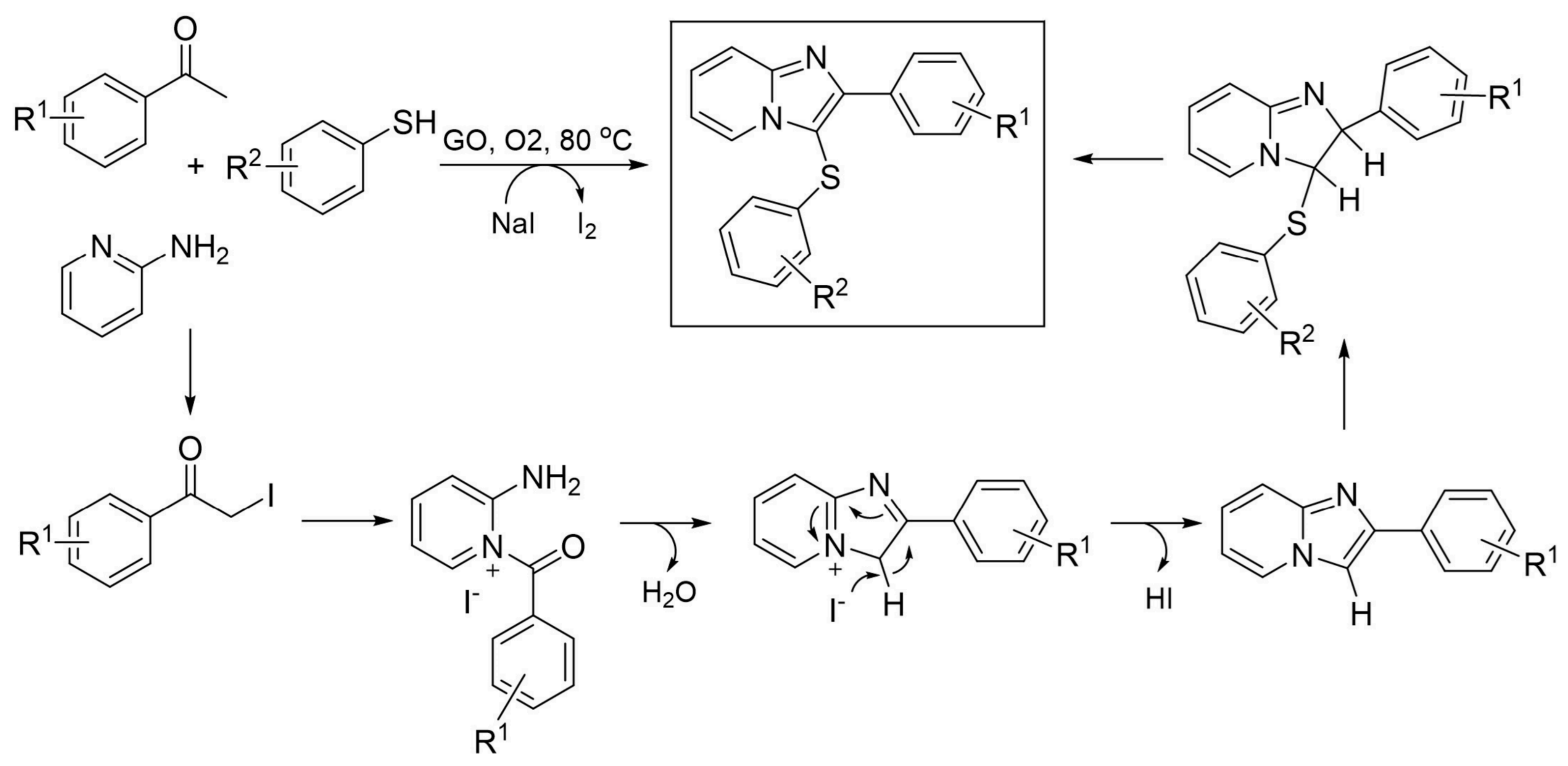
Proposed reaction mechanism for imidazopyridine synthesis catalyzed by GO.

Interestingly, Dhopte et al. [88] recognized GO as a catalyst with a dual function, with the acid catalyst and oxidizing agent in benzimidazole and benzothiazole synthesis at high yields (up to 75% after 3–5 h), from *o*-phenylenediamine or *o*-aminothio-phenol with aromatic aldehydes, in MeOH, upon ultrasound irradiation at 35 °C (150 W at 33 kHz), under aerobic conditions (Scheme 5). In this case, the use of water produced a decrease in the reaction yield due to the low reactant solubility. The authors experimentally confirmed the reaction mechanism by monitoring this via gas chromatography. Firstly, the imination reaction between reactants occurred, catalyzed by acid sites, with subsequent oxidative cyclization assisted by GO, which was reduced partially in situ, although it can be regenerated by re-oxidizing. Remarkably, the reaction takes place even in the absence of molecular oxygen, demonstrating the oxidizing capability of GO. In a similar context, Kundu and Basu [89] reported a methodology for the synthesis of imidazo [1,2–a]pyridines (81–93%, 6–9 h), and 3–sulfenylimidazo [1,2–a]pyridines (70–89%, 10–15 h) from 2–aminopyridines, acetophenone, and thiol catalyzed by GO, in toluene, at 80 °C, in the presence of NaI as additive. In this case, the authors claimed the role of GO to transform NaI to I_2_, but it was also involved in the last oxidation step for the corresponding 3–sulfenylimidazopyridine (Scheme 6).

**Scheme 7:**
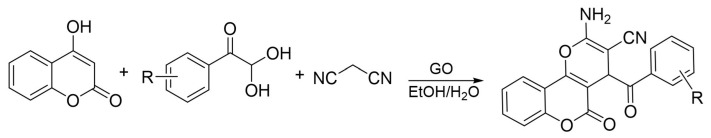
Synthesis of pyranocoumarins catalyzed by GO at 80 °C.

Khodabakhshi et al. [90] reported the three-component synthesis of pyranocoumarins from 4-hydroxycoumarin with aryl glyoxals and malononitrile catalyzed by GO, in EtOH/water (1:1) as the optimum solvent, at 80 °C (Scheme 7). The methodology is tolerant to a variety of functional groups (Ar = Ph, 4-F-C_6_H_4_, 4-Cl-C_6_H_4_, 4-Br-C_6_H_4_, 4-NO_2_-C_6_H_4_, 4-MeO-C_6_H_4_, naphthyl, etc.), affording the corresponding pyranocoumarins at a high yield after short reaction times (up to 85% after 60–85 min). Interestingly, typical basic catalysts such as NaOH or Na_2_CO_3_ ended up being totally ineffective while GO successfully promoted the synthesis in the presence of differently substituted aryl glyoxals. Although there is no clear evidence concerning the reaction mechanism, the most probable active sites in GO seem to be acidic and carbonyl functions, with hydrogen bonding being probably responsible for the aryl glyoxal activation.

**Scheme 8:**
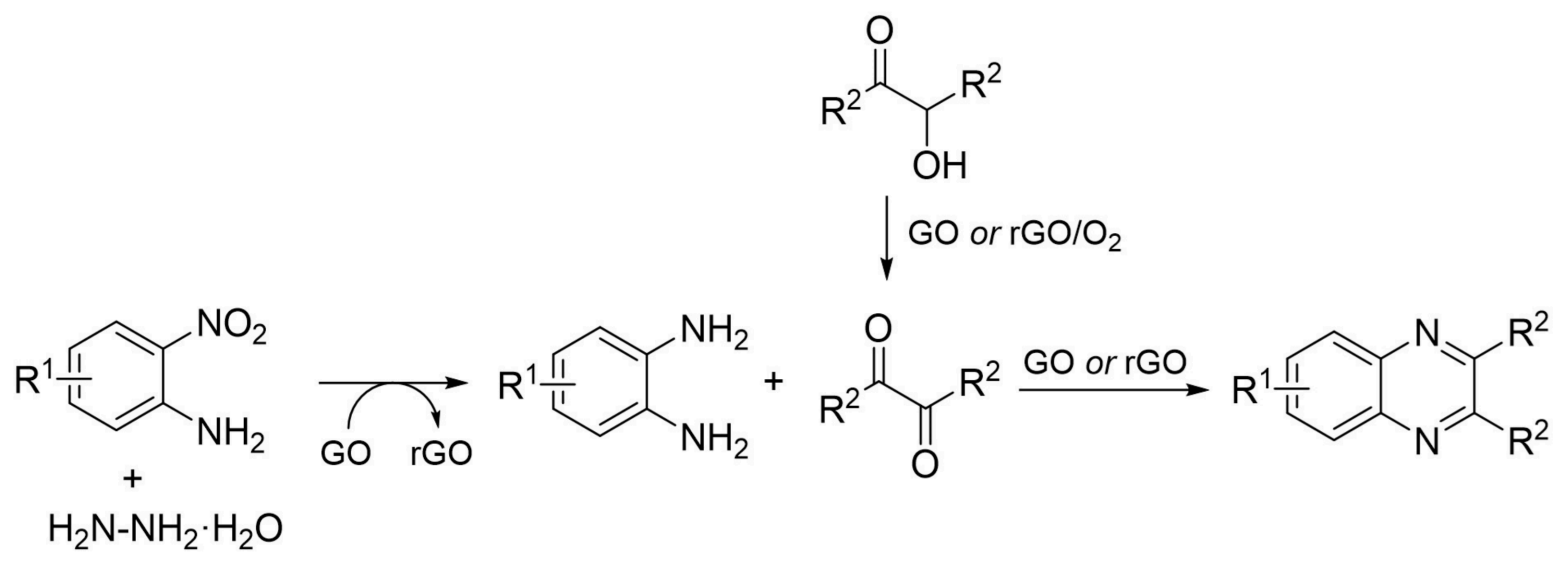
Synthesis of quinoxalines from 2-nitroanilines and benzyls, bezoins, or 1,2-diols catalyzed by GO.

An interesting synthetic strategy for the synthesis of quinoxalines, at high yields (up to 83%), from 2-nitroanilines and benzyls, bezoins, or 1,2-diols promoted by GO, has been reported by Roy et al. [91] (Scheme 8). Its relevance relies on using a metal-free catalyst assisting with redox and condensation reactions. Since the developed methodology includes the use of the hydrazine hydrate excess in large amounts, as a hydrogen donor, which can react with benzyls to form the corresponding hydrazones, the reaction did not follow a conventional multicomponent synthesis protocol. In this case, the process involved two separated reaction steps in one pot, consisting of the initial reduction of nitroanilines with hydrazine hydrate catalyzed by GO until disappearing (at 100 °C), followed by adding the carbonyl compound precursor for cyclization (at 60 or 80 °C). Although the role of GO on the first reduction of nitroanilines is unclear, the authors proposed that GO is firstly reduced to rGO, acting as an electron conductor through the carbon surface area, favoring the hydrogen releasing from hydrazine. GO would be involved in the oxidation reaction of benzoin to benzyl, as well as in the condensation reaction with the formed 1,2-diamines to give quinoxalines with high yields.

**Scheme 9:**
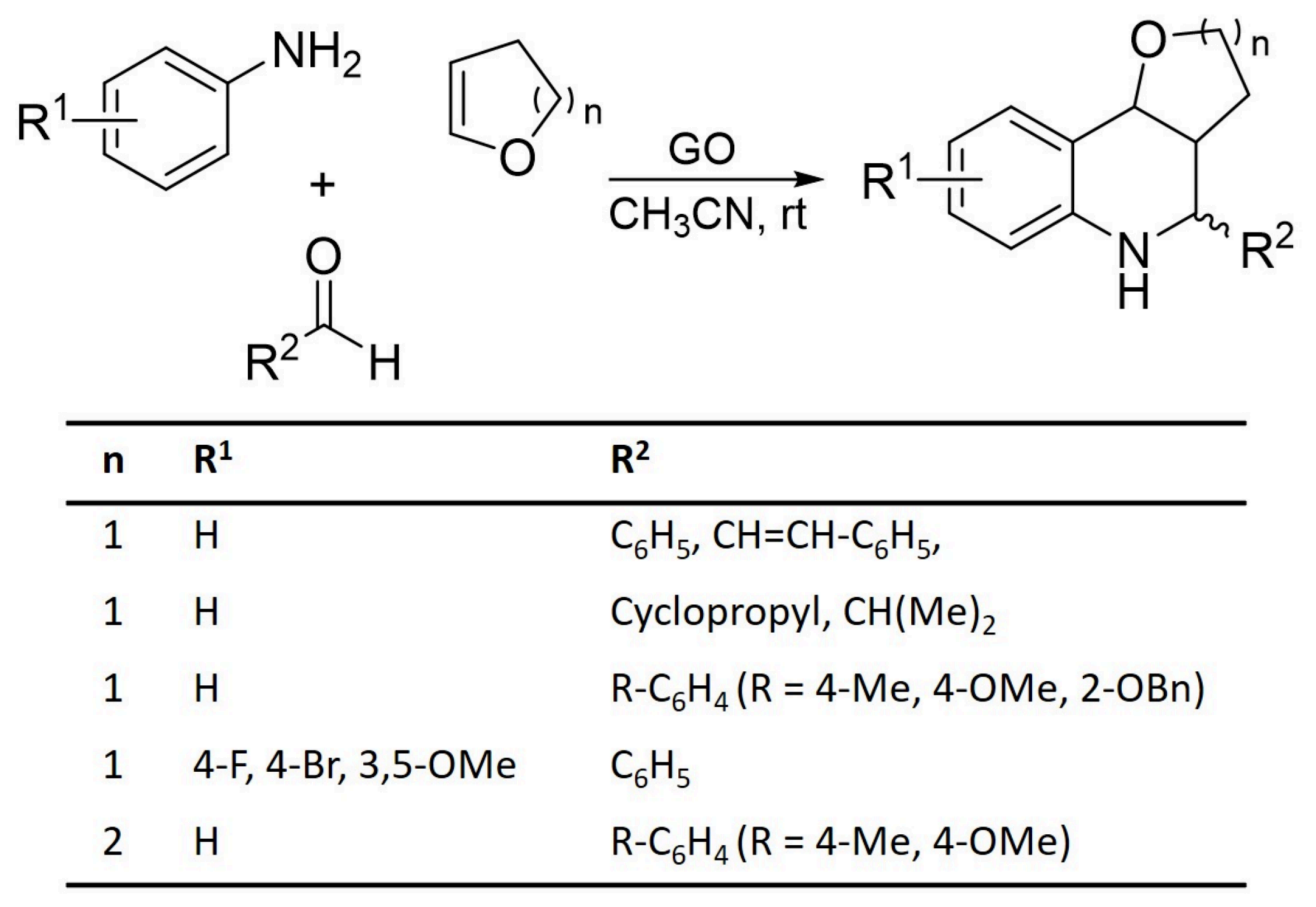
Synthesis of 2,3-disubstituted quinolines through Povarov multicomponent reaction.

Similarly, using the above-mentioned dual capacity of GO as both an acid but also an oxidant, Caputo et al. [92] reported the synthesis of 2,3-disubstituted quinolines, in CH_3_CN at room temperature, through the Povarov multicomponent reaction, obtained as mixtures of endo/exo isomers. Imination and the aza-Diels–Alder reaction would be promoted by acid catalytic sites with the final oxidation of intermediated tetrahydroquinolines to quinolines, observing the overall reduction of GO and its covalent chemical modification with reactants and products, confirmed by XPS and NMR, both notably reducing the catalytic activity (Scheme 9). The authors highlight that the Lewis acidity on GO, attributed to the basal plane epoxide ring opening, could be behind the observed catalytic performance.

##### P- and S-Doped GO

OSGs at the GO surface allow easy covalent functionalization with different functional groups in nature, often P- or S-containing groups, conferring a high hydrophilicity which increases the long-term stability of the dispersed sample. However, only a few reports concerning phosphonated or sulfonated GO are known, probably due to the difficult control of the functionalization. In this context, P-doped GO has been successfully applied as a reusable catalyst in the multicomponent synthesis of pyrano [2,3-c]pyrazoles (yield = 80–90%, 15 min), from hydrazines, ethyl acetoacetate, malononitrile, and benzaldehydes, in water at reflux [93]. Ghafuri and Talebi [94] applied the phosphorylated GO (PGO), obtained from GO by using different phosphorylation agents such as H_3_PO_4_, POCl_3_, and PCl_3_, (P_2_O_5_), [P(OEt)_3_], for benzimidazole synthesis at high yields (up to 87%, 45–90 min), in EtOH, at room temperature. PGO showed total selectivity to the corresponding benzimidazoles, making it an advantageous catalyst compared with pristine GO. Similarly, sulfonated GO (SGO), prepared by the hydrothermal sulfonation of rGO using fuming sulfuric acid at 180 °C, was found to be a highly efficient catalyst for the Peckmann reaction of resorcinol with ethyl acetoacetate to produce coumarins [95].

#### 3.2.2. Miscellaneous

**Scheme 10:**
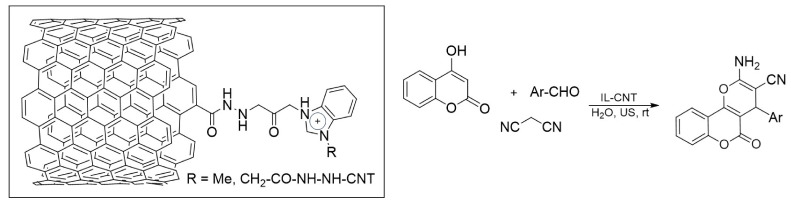
Three component synthesis of chromene derivatives catalyzed by IL@CNTs.

Advanced metal-free carbon-based materials, such as CNTs or carbon dots, have barely been used for the one-pot synthesis of heterocycles. In this sense, sophisticated strategies are reported to have a specific effect. Mayank et al. [96] covalently modified the carbon surface of CNTs by functionalization with different ionic liquids (ILs) in such a manner that the surface was positively charged, notably increasing the surface area and pore volumes. These materials efficiently catalyzed chromene synthesis under ultrasound irradiation, using water as the solvent, with the functionalities contributing to the catalytic activity (Scheme 10). By following another synthetic approach, carbon dots encapsulated within IL micelles were reported to form a carbon dot–colloidal reaction system that, by combining the catalytic activity of carbon dots with the solubilization and confinement effects of micelles, enables efficient organic transformations in aqueous media. In this work, the carbon dots were synthesized by a bottom–up hydrothermal method using citric acid as a carbon precursor and used in the catalytic system for the one-pot multicomponent synthesis of 4*H*-chromene derivatives. The catalytic activity was attributed to the surface functional –COOH and –OH groups present at the carbon dots’ surface [97].

**Scheme 11:**
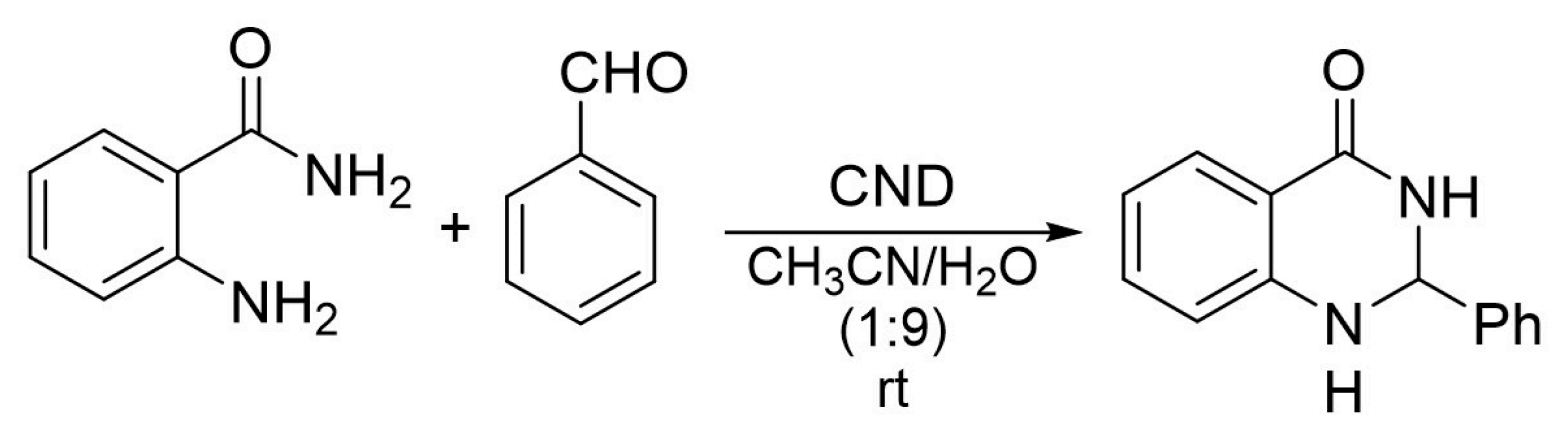
Synthesis of quinazolinones from 2-aminobenzamide and aldehydes catalyzed by acid CND. Copyright © 2017, American Chemical Society [98].

Majumdar et al. [98] prepared fluorescent carbon dots, prepared from β-carotene as a carbon source under hydrothermal conditions at 180 °C, useful for the efficient synthesis of quinazolinones from 2-aminobenzamide and differently substituted aldehydes (Scheme 11). Considering the worst yields obtained when using the derived reduced carbon dots, the authors claimed the involvement of the −COOH functions promote the reaction.

## 4. Conclusions

Given the necessity for sustainability, Green Chemistry is promoting an inevitable transition toward the use of metal-free catalysts. The implementation of metal-free carbon-based catalysts represents a paradigm shift in Fine Chemistry, where the structure–function interdependence is intrinsically governed by the nature of the precursor. Note that the term ‘metal-free carbon catalyst’ implies carbon catalysts with the absence of any metal impurities, experimentally corroborated, in a task that is sometimes difficult to achieve, supposing a constant problem, e.g., carbon catalysts from biomass resources and other waste, or even CNTs whose synthesis is catalyzed by metals. While the use of pure substances to synthesize carbon-based catalysts, from carbon gels, CNTs, and COFs, among others, facilitate the functionalization on the surface and exhibit superior kinetic properties, they are constrained by complex synthetic strategies and scalability barriers. The valorization of waste, particularly of plastic ones, is emerging as a strategic and sustainable pathway, allowing the obtention of carbonaceous materials with a high inherent purity. However, the optimization of the conversion methodologies is required in order to mitigate the heterogeneity and ensure a consistent catalytic performance at the industrial scale.

Not only is the importance of determining the catalytic sites directly implied on the chemical synthesis unquestionable, but also, in the development of more effective and improved catalytic systems. Currently, the available characterization and operando techniques for the knowledge of the catalysts in combination with theoretical calculations and machine-learning techniques undoubtedly contribute to knowing the processes, as well as the design of new generations of catalysts—in this case, of metal-free carbon catalysts, with improved properties.

Considering the importance of heterocyclic systems in the pharmaceutical industry and that the adoption of green practices is currently almost due to strict environmental regulations, the use of metal-free carbon catalysts promoting these transformations can be considered a valuable alternative in terms of sustainability. As mentioned above, the most investigated metal-free carbon catalyst is GO, catalyzing both condensation and oxidative and reduction reactions. Advantageously, its great hydrophilic character allows for a high dispersion in water or alcohols, making it an interesting catalyst operating under greener conditions. Of especial interest is that GO can act as a benign oxidant, as demonstrated in a few reports. However, considering the high functionalization degree of GO, the identification of the nature of active sites is a difficult task [99]. Considering that the carbon surface is very complex due to the presence of a great diversity of functional groups, the development of methodologies that allow the control of the formation of specific active sites represents a challenge still unsolved. The versatility of these materials constitutes both an advantage and a challenge: although a tailored specific design of active sites is achievable, isolating a single type of active site within the catalyst remains challenging.

## Figures and Tables

**Table 1:** Heterocycle synthesis catalyzed by GO.

Heterocycle	Substituents	Catalyst	Solvent	T/t (°C/h)	Yield (%)	Ref.
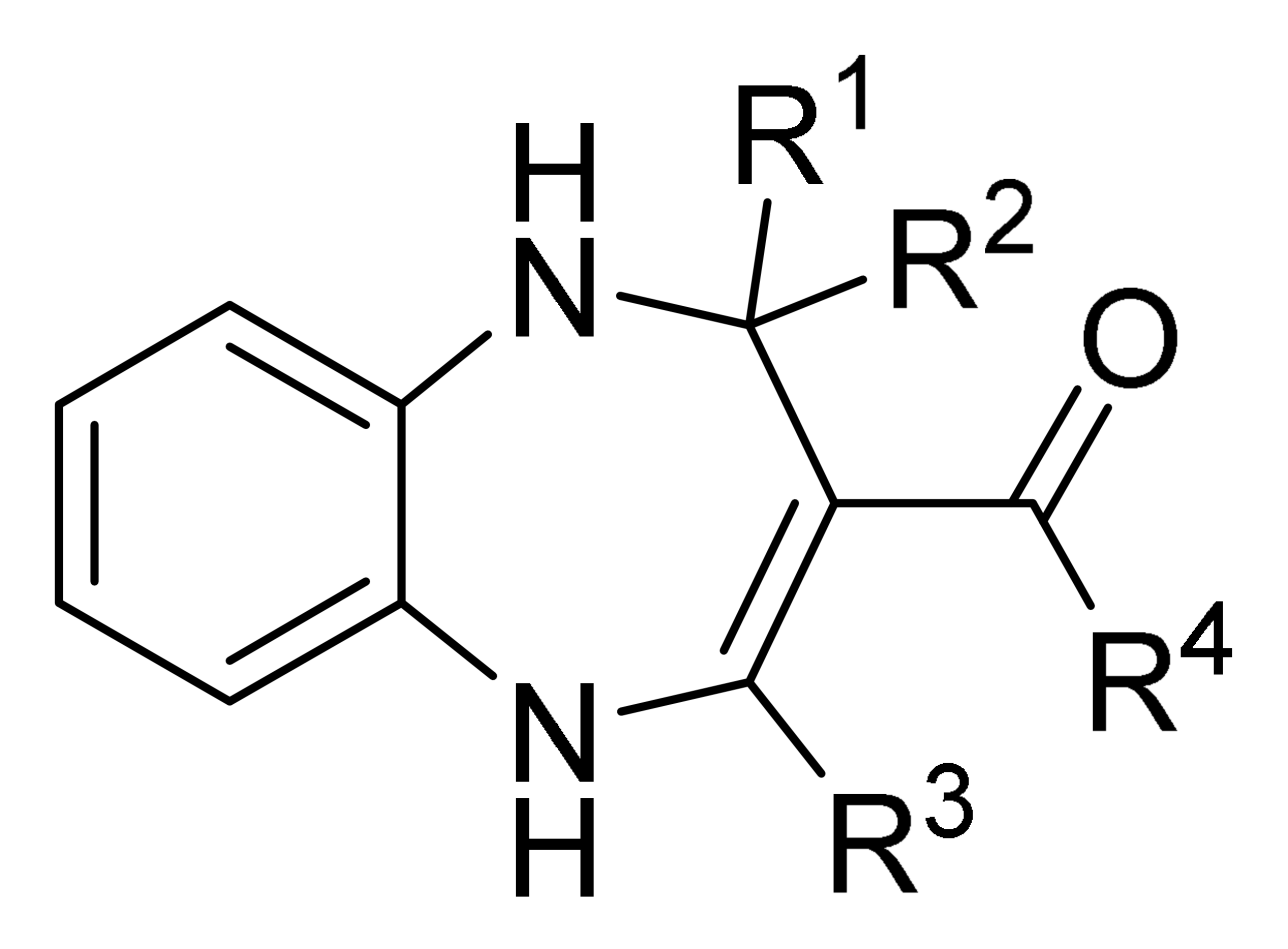	R^1^ = Ph; R^2^ = H R^3^–R^4^ = –CH_2_-C(Me)_2_-CH_2_–	GO PEG-SO_3_H	H_2_O H_2_O	70/0.5 100/3	95 55	[81]
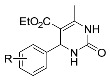	R = H, 4-OMe, 4-Me, 4-Cl, 4-Br 2- Cl, 2-NO_2_,	GO	Solvent-free	rt (US)/2–3	76–90	[82]
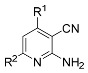	R^1^ = 4-MeC_6_H_4_ R^2^ = C_6_H_5_	GO BEA Silica TiO_2_	H_2_O H_2_O H_2_O H_2_O	80/5 80/5 80/5 80/5	90 66 49 40	[83]
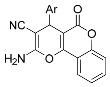	Ar = C_6_H_5_	GO MgO KF-Al_2_O_3_	H_2_O/EtOH 1:1 EtOH EtOH	60/14 min Reflux/32 min Reflux/4	95 89 90	[84]
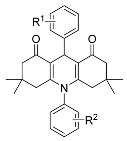	R^1^ = H, 4-Cl, 4-Br, 4-F, 4-Me, 4-OMe, 4-NO_2_, 3-NO_2_ R^2^ = 4-Cl, 4-Br	GO	DMF	100/60–120	91–94	[85]
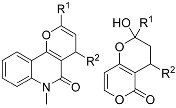	R^1^ = Ph; R^2^ = 4-C_6_H_4_	GO nanosheet	H_2_O	80/3	95, 90	[86]
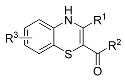	R^1^ = Me, Ph, *t*-Bu, OCH_3_, etc. R^2^ = Me, Ph, *t*-Bu, CN etc. R3 = H, 6-Cl, 6-CF_3_	GO	Solvent-free	rt/8, 24	75–88	[87]

## Data Availability

No new data were created or analyzed in this study. Data sharing is not applicable to this article.
